# One-year follow-up of patients with long-lasting post-herpetic neuralgia

**DOI:** 10.1186/s12879-014-0556-6

**Published:** 2014-11-01

**Authors:** Francesca Pica, Antonio Gatti, Marco Divizia, Marzia Lazzari, Marco Ciotti, Alessandro Fabrizio Sabato, Antonio Volpi

**Affiliations:** Dipartimento di Medicina Sperimentale e Chirurgia, Università di Roma Tor Vergata, Via Montpellier, 1, Rome, 00133 Italy; Emergency Care, Department of Intensive Care, Pain Medicine, and Anaesthesiology, Policlinico Tor Vergata, University of Tor Vergata, Rome, Italy; Fondazione Policlinico Tor Vergata, Via Oxford 83, Rome, Italy; Dipartimento di Scienze Cliniche e Medicina Traslazionale, Università di Roma Tor Vergata, Via Montpellier, 1, 00133 Rome, Italy

**Keywords:** Chronic pain, Herpes zoster (HZ), Post-herpetic neuralgia (PHN), Quality of life

## Abstract

**Background:**

Recent information on epidemiology and management of post-herpetic neuralgia (PHN), a painful complication of zoster, is scarce.

**Methods:**

This study was conducted at the Pain Clinic of the Policlinico Tor Vergata, Rome, Italy, on eighty-five immunocompetent patients with a clinical diagnosis of PHN. At enrollment (time 0, T0), the patients were interviewed by physicians to obtain demographic data and information about their zoster clinical history and underwent a blood test for VZV-DNA research. DN4 and SF-12 questionnaires were used to assess the neuropathic nature of pain and the overall health status, respectively. A one-year follow-up was planned for enrolled cases, who were visited at regular intervals of at least 3 months.

**Results:**

At T0 all the patients were at least 6 months from the episode of acute zoster and still presented with intense pain (mean VAS =6.7; mean DN4 = 5.7). Using antivirals within 72 hours from the rash onset was associated to a significant reduction of pain at T0 (*p* = 0.006 *vs* untreated patients). Only 2.6% of patients treated with antivirals during acute zoster but 18.6% of the untreated ones presented with neuropathic pain at T12 (*p* =0.007), even though the two groups were similar at T0. VZV-DNA was found in 5 out of the 50 available blood samples. At the last follow-up visit, PCS and MCS scores of the PHN patients were found to be recovered over those of the historical age-matched healthy controls. Undesirable side effects of analgesic therapies were observed in 15.3 to 28.8% of the patients.

**Conclusions:**

Patients who six months after acute zoster still have significant neuropathic pain, have a high probability of suffering from chronic pain in the subsequent months/years. The initial antiviral treatment has a significant impact on the pain. Current strategies of analgesic therapy are effective to achieve relief of pain in PHN patients, but they are burdened with heavy and undesirable side effects.

**Electronic supplementary material:**

The online version of this article (doi:10.1186/s12879-014-0556-6) contains supplementary material, which is available to authorized users.

## Background

Post-herpetic neuralgia (PHN) is the most challenging and debilitating complication of herpes zoster (HZ) in immunocompetent hosts. It is characterized by constant or intermittent burning, itching or aching, with paroxysmal or lancinating pain. Other primary characteristic, such as numbness, tingling and allodynia, also contribute to the burden of PHN. Pain intensity at the rash onset, age, rash severity, length of prodromal pain and cranial localization are more frequently reported as predictors of PHN [1]-[14]. Several issues concerning diagnosis, prediction and prevention of PHN need, however, to be clarified in view of recent contributions [15]-[20].

Despite advances in antiviral therapy during acute HZ and the more recent introduction of vaccination against Varicella-zoster virus (VZV) [21], PHN continues to be a significant clinical problem, with up to 25% of patients over 60 years developing persistent neuropathic pain following acute HZ [22]-[24]. The estimated incidence of PHN varies with its definition [25]-[30]. PHN can persist, in some individuals, for months or years after the HZ rash has healed, causing suffering for the patient and a burden of economic cost on patient, care givers, and healthcare providers. Studies vary widely in the reporting of the duration of persistent pain [31]. Helgason et al. [32] found that of 13 subjects with persistent pain 12 months after HZ, 6 still reported pain after 6.3 years. In one study of patients aged over 65 years, the mean duration of pain was 3.3 years, and ranged from 3 months to more than 10 years [33]. A few recent prospective studies report on the persistence of symptoms up to and over 12 months after HZ onset. In detail, Bouhassira et al. [34] reported the presence of zoster-related pain in 6% of 1032 patients 12 months after HZ. McKendrick et al. [35] found that of 158 subjects assessed 9 years after HZ, 21% had experienced any pain during the past year; of these, 47% had been pain-free at the time of discharge from the acute study. This is the reason why, in the last years, PHN is emerging as a preferred clinical trial model for chronic neuropathic pain [36].

The treatment of PHN is presently based on a well characterized array of drugs and drug associations with proved efficacy, including tricyclic antidepressants, the antiepileptic drug gabapentin, pregabalin and opioids, with some evidence also for topical lidocaine [36]. It remains still unsatisfactory, however, in a substantial proportion of patients, especially those with many comorbidities and more intense pain at HZ presentation [36].

Acute HZ and consequently PHN particularly afflicts the immunocompromised and elderly patients, a fact that has serious implications for health-care delivery in the context of ageing populations in the developed world and the worldwide spread of HIV disease. Left untreated, PHN can become a severe and debilitating condition affecting all aspects of a patient’s life [37].

The present report focus on the actual impact of diagnosis and management of PHN. In detail, the correlations existing between demographic and clinical characteristics of patients, the possible utility of VZV-DNA research in blood, and the efficacy of treatments for pain relief on the quality of life of PHN patients have been examined.

## Methods

### Study population and design

Patients with a clinically diagnosed PHN, with pain over 3 VAS for at least 6 months after the onset of acute zoster, were consecutively enrolled at the Pain Clinic of the Policlinico Tor Vergata, Rome, Italy, and examined and treated between January 1, 2009 and November 15, 2010. The study was submitted to and received approval by the Independent Ethical Committee of the University of Rome “Tor Vergata”.

Eighty-five immunocompetent patients with PHN gave written informed consent to be included in the study. They were interviewed by physicians to obtain demographic data (i.e. name, age, sex, race/ethnicity), memory of primary VZV infection and HZ history [presence of a prodrome (defined as pain before rash onset) and its duration (range 1 - over 3 days), extent of rash (rash involving 1, 2, or more than two dermatomes) and its localization, intensity of pain, presence of abnormal sensations (including tingle and allodynia) and/or itch, and antiviral therapy]. All data obtained from the interviews were recorded in database files.

Participants in the study also were asked to undergo a blood test for VZV DNA research at the time of enrollment. Blood sampling (5 ml whole blood per patient) was performed after an overnight fast. Whole blood samples were collected and DNA was extracted using a QIAamp DNA blood mini kit (Qiagen Ltd., United Kingdom) according to the manufacturer’s instructions. The eluted DNA was stored at -20°C. Detection and quantification of VZV DNA were performed by using a VZV Q-PCR Alert kit (Nanogen Advanced Diagnostics, Torino, Italy). The cut-off of the method was 56 viral copies/ml and below this level it is able to detect up to 10 copies/ml of virus, if present.

Different tools were used to quantify and qualify pain in the enrolled PHN patients. Briefly, the intensity of pain was evaluated using a visual analog scale (VAS) of 10 cm in length that was graduated from “0” to “10,” where “0” represented no pain and “10” the most unbearable pain.

The DN4 questionnaire (which stands for Douleur Neuropathique en 4 Questions) was used to estimate the probability of neuropathic pain in our PHN patients. It consists of 10 items subdivided into two parts: sensory descriptors (seven items) and signs relating to the sensory examination (three items). The presence of neuropathic pain was taken to be the dependent variable and needed to reach a score of at least 4 out 10, while non-neuropathic pain presented scores of less than 4 out 10. This questionnaire has been well validated in a number of studies [37]-[39].

The SF-12 questionnaire was also used in the medical interviews [40]. It is a generic short form health survey developed in the USA from the original SF-36, which produces two summary measures evaluating physical and mental self-perceived health that are interchangeable with those from the SF-36. SF-12 has been successfully tested in nine Western European countries, included Italy, on large samples of the general population, where it has proved its brevity, comprehensiveness, reliability, validity and cross-cultural applicability [40],[41].

A one-year follow-up was planned for all the enrolled cases, who were visited at least every 3 months. Patients were subjected to drug therapy according to the protocols currently in use at international level [42],[43]. Treatment of pain was chosen on the basis of the international specific guidelines [36] paying particular attention to the presence of eventual comorbidities, drug interactions with drugs already used in the therapy and potential side effects.

### Statistical analysis

Statistical analysis and data processing were performed using SPSS software – version 20 for Windows™. All two-sided statistical tests were performed with a 5% significance level. Quantitative variables were analysed by descriptive statistics including mean values, standard deviation, median, minimum and maximum. Clinical and demographic characteristics were analysed by Chi-square test and Analysis of variance (ANOVA).

## Results

### Study population

The demographic and clinic characteristics of the PHN patients enrolled in the study are listed in Table 1. Overall, the group consisted of 85 immunocompetent individuals (50 female and 35 male) who had received a clinical diagnosis of HZ at least 6 months before the enrollment, and whose median age was 74.5 years (range 47-92) for female and 77 years (range 41-90) for male. All of them were Caucasian Italians and out-patient at the Pain Clinic of the Policlinico Tor Vergata, Rome, Italy.

**Table 1:** **Demographic characteristics and herpes zoster history of patients with post-herpetic neuralgia**

	Female	Male
**Number**	**50**	**35**
**Age**	N (%)	N (%)
< 50	2 (4)	1 (3)
51-60	4 (8)	3 (9)
61-70	9 (18)	2 (6)
71-80	18 (36)	13 (27)
>80	16 (32)	16 (46)
Median (range)	74.5 (47-92)	77 (41-90)
**Prodromal pain**		
< 1 day	29 (58)	27 (77)
1-3 days	10 (20)	2 (6)
3 days	11 (22)	6 (17)
**Localization of rash**		
Cranial	9 (18)	10 (29)
Cervical	6 (12)	4 (11)
Thoracic	24 (48)	13 (37)
Lumbar	11 (22)	8 (23)
**Severity of rash**		
Mild	7 (14)	4 (11)
Moderate	22 (44)	14 (40)
Intense	16 (32)	15 (43)
Severe	5 (10)	2 (6)
**Therapy**		
No	23 (46)	20 (57)
Yes	27 (54)	15 (43)

The information on age, gender, HZ diagnosis and history, the overall health status and the immune status were collected by medical interview and visit at the time of enrollment in the study. Immune status was determined on the basis of the absence of specific conditions or treatments, i.e. active cancer, solid organ transplant, AIDS, chronic corticosteroid use, or other immunosuppressive treatments for autoimmune diseases or other conditions.

### Characteristics and correlates of pain

All the patients, in both gender categories, reported to have had prodromal pain and abnormal sensations in the days (from 1 to 3) preceding the HZ rash onset. The rash localization was thoracic in 37 patients and cranial, cervical or lumbar in 19, 10 and 19 patients, respectively. The intensity of rash was moderate or severe in the 87% of patients and mild in the remaining 13%. Only one half of the patients had received specific antiviral therapy prescription at the time of the HZ episode.

No statistically significant difference between the two gender groups in relation to the different parameters showed in Table 1 was found, with the only exception of the localization of rash (*Chi-square*: p < 0.03).

**Figure 1:**
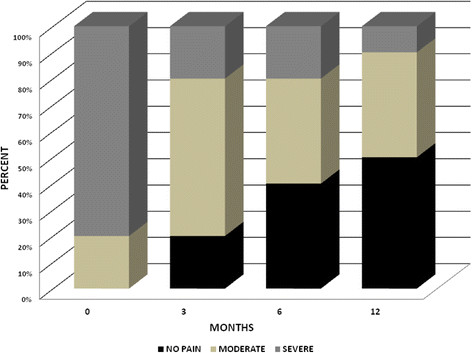
**Intensity of pain in patients with post-herpetic neuralgia during the follow-up period.**

Intense or very intense pain at the time of enrollment (*i.e.* time 0, T0) was reported by the majority of the patients (Figure 1). The patients reporting intense or very intense pain at T0 were equally distributed in the two gender categories (*p* =0.22).

The median duration of pain (time from the rash onset), which was equal to 9 months (range 6-150) in the total population sample, was found to be longer for women than men, *i.e.* 12 months (range: 6-150) and 6 months (range: 6-72), respectively ( *p* = 0.067*).*

The measures of the intensity and quality of pain at the time of enrollment and during the follow-up period are reported in Table 2, where patients with PHN have been grouped in three main age groups. The neuropathic nature of pain of participants in the study was assessed by the positivity of DN4 questionnaire, with scores greater than 4 in all the patients examined (Table 2). No difference by age group in relation to the parameter “pain” was found in our population sample (Table 2).

**Table 2:** **Characteristics of pain in patients with post-herpetic neuralgia**

	Age			
	55-64 (15 pts) Mean ± SD	65-74 (23 pts) Mean ± SD	>75 (47 pts) Mean ± SD	P*
**VAS at presentation**	6.80 (±1.265)	6.83 (±1.230)	6.62 (±1.649)	0.879
**VAS at 3 Months**	4.80 (±1.1568)	4.00 (±2.412)	3.96 (±1.769)	0.355
**VAS at 6 Months**	3.67 (±2.690)	3.39 (±2.331)	3.64 (±2.110)	0.933
**VAS at 12 Months**	2.27 (±1.944)	2.28 (±1.864)	2.85 (±2.167)	0.487
**DN4 at presentation**	5.80 (±1.265)	5. 48 (±1.163)	5.13 (±1.154)	0.111
**DN4 at 12 Months**	1.60 (±1.298)	1.35 (±1.434)	1.45 (±1.4)	0.776

*Statistical analysis of differences by Kruskal-Wallis Test.

The use of antiviral therapy (i.e. Aciclovir, Valaciclovir, Famciclovir or Brivudin), starting within 72 hours from the rash onset, had been capable of inducing a significant reduction of pain (VAS average) at the time of enrollment at the Pain Clinic (T0) (*p* = 0.006 *versus* untreated patients). Further analyses confirmed a significant risk of having greater pain among patients who had not received antiviral therapy (Table 3). Moreover, only 2.6 percent of patients treated with antivirals during acute zoster but 18.6 percent of the untreated ones presented with neuropathic pain at T12 (*p* =0.007), even though no difference among the two groups had been observed at the time of enrollment (T0).

**Table 3:** **Relative risk (RR) of having VAS lower than 5 for patients with post-herpetic neuralgia, who had been treated with Antivirals within 72 hours from the rash onset**

	*p*	*RR (CI 95%)*
**VAS <5 at T0**	NS	0.86 (0.53-0.41)
**VAS <5 at T3**	NS	0.67 (0.34-1.3)
**VAS <5 at T6**	0.017	0.42 (0.17-1.0)
**VAS <5 at T12**	0.05	0.27 (0.04-1.7)

The efficacy of treatments for pain relief given to the patients during the overall period of examination, was demonstrated by a significant reduction of pain (VAS average) in the patients with PHN at all the times tested (p <0.001), without significant differences among different age groups (Table 2). However, several patients reported the occurrence of adverse effects related to these treatments [i.e. 19/85 (22,4%) at 3 months, 13/85 (15,3%) at 6 months and 24/85 (28,2%) at 12 months]. The main reported side effects were drowsiness, dizziness, ataxia, mild peripheral edema, and a worsening of cognitive impairment in elderly patients, in relation to treatments with the anticonvulsants, gabapentin and pregabalin; but also anticholinergic side effects such as sedation, orthostatic hypotension, cognitive decline, and constipation related to tricyclic antidepressant (TCA), or nausea, vomiting, dizziness, constipation, drowsiness and headache related to tramadol.

### PCS and MCS scores

The Physical and Mental Health Composite Scale (PCS and MCS) scores of the patients enrolled in the study were measured by means of the SF-12 questionnaire. As expected, they varied as a function of the patients’ age and were found to be lower in older individuals than in the younger ones (Table 4).

**Table 4:** **Physical and mental health composite scale scores in patients with post-herpetic neuralgia**

	55-64 (15 pts) Mean (±SD)	65-74 (23 pts) Mean (±SD)	>75 (47 pts) Mean (±SD)
**MCS at the first visit**	34.86 ± 5.3	34.00 ± 5.8	33.1 ± 8.0
Historical controls*	*49.03 ± 10.41*	*47.79 ± 11.10*	*45.38 ± 12.09*
**MCS at 3 Months**	45.00 ± 8.4	41.2 ± 7.0	38.9 ± 8.6
**MCS at 6 Months**	47.00 ± 9.0	45.00 ± 9.1	42.6 ± 10.0
**MCS at 12 Months**	55.6 ± 3.0	53.3 ± 4.7	51.0 ± 5.6
**PCS at the first visit**	37.9 ± 7.6	30.7 ± 5.2	29.6 ± 4.9
Historical controls*	*48.05 ± 9.52*	*44.04 ± 10.71*	*37.85 ± 11.60*
**PCS at 3 Months**	39.3 ± 6.9	37.9 ± 5.4	34.5 ± 6.4
**PCS at 6 Months**	45.0 ± 7.4	41.5 ± 7.9	36.8 ± 7.8
**PCS at 12 Months**	53.1 ± 5.9	49.4 ± 5.2	44.0 ± 7.7

*Apolone G et al. QUESTIONARIO SULLO STATO DI SALUTE SF-12 - Versione italiana IRFMN 2005. Copyright 2001 Istituto di Ricerche Farmacologiche Mario Negri, Milano.

As shown in the Table 4, the PCS and MCS mean scores of our patients at the time of enrollment in the study were found to be sensibly lower than those measured in a large Italian population sample of healthy individuals [40], who were used as an historical age-matched control, thus confirming the devastating effects of PHN on the patients’quality of life in all the age groups.

At the time of enrollment, PCS mean scores of our patients were found to be lower in older patients than in the younger ones, whereas MCS mean scores appeared to be quite similar (Table 4). The effectiveness of the treatments used to relieve pain during follow-up was demonstrated in all the PHN patients studied, through the observation of an apparent increase in both PCS and MCS scores, which were found even higher than those of the historical control (Table 4). Finally, in our study PCS and MCS mean scores did not differ depending on the gender of the patients examined (data not shown).

### VZV-DNA positivity in peripheral blood of patients with PHN

Five out of the 50 available blood samples from the total of 84 PHN patients enrolled in this study were found to be positive for VZV DNA. Among them were 3 males (66, 81 and 88 years-old) and 2 females (48 and 75 years-old). Four of them had been affected by thoracic zoster, whereas the fifth was a 48 y-o woman who has presented with trigeminal zoster. The demographic and clinic characteristics of VZV DNA-positive PHN patients are summarized in the Table 5.

**Table 5:** **Demographic and clinical characteristics of patients with post-herpetic neuralgia and positive VZV viremia**

	Patient n.1 Male, 66 y-o	Patient n.2 Female, 75 y-o	Patient n.3 Male, 88 y-o	Patient n.4 Female, 48 y-o	Patient n.5 Male, 81 y-o
Prodromal pain	< 1 day	< 1 day	< 1 day	< 1 day	< 1 day
Rash onset	6 months	80 months	7 months	60 months	36 months
Rash localization	Thoracic	Thoracic	Thoracic	Trigeminal	Thoracic
Rash intensity	Severe	Intense	Moderate	Moderate	Moderate
Antiviral therapy	No	No	Yes	No	No
VAS (T0)	8	6	7	8	5
DN4 (T0)	7	8	4	4	4

## Discussion

This study confirms and extends previous observations about the natural history of zoster pain in a cohort of eighty-five consecutive immunocompetent PHN patients, who were followed prospectively after their admission at the Pain Clinic, Policlinico Tor Vergata, Rome, Italy, to receive proper medical therapy for pain relief.

At the time of study entry all the PHN patients were at least 6 months from the episode of acute zoster and still presented with intense or very intense pain (VAS >6), whose neuropathic nature was confirmed by the positivity of DN4 questionnaire, with scores greater than 4 in all the patients examined.

Our results clearly indicate that patients who still have significant neuropathic pain (VAS ≥ 6, DN4 > 4) 6 months after the acute episode of zoster , have a high probability of suffering from chronic pain in the subsequent months/years. This finding is in accordance with data from the international literature, since, on the basis of recently published prospective studies, six months is just emerging as a reliable time-threshold to discriminate, in the context of patients with PHN, between those with a more favorable or poor prognosis [44]. Consistently, Reda and coll. have shown that in a population of immunocompetent patients at high risk of PHN (for both older age and higher pain intensity), no subjects who were pain free at 6 months showed a recurrence of pain on subsequent visits [44].

We had shown previously that older age, greater acute pain intensity, greater rash extent and longer duration of prodromal pain are independent risk factors in the development of post-herpetic neuralgia, by analyzing the relationship between baseline and 6-month follow-up data in a sample of 219 Italian immunocompetent herpes zoster patients [15]. Even considering the possibility of recall bias, which is inherent to any study in which subjects are asked to recall events that may have occurred months or even years before [16], the results of the present study clearly confirm previous observation that severity of disease on acute herpes zoster presentation and older age are the best indicators of developing post-herpetic neuralgia.

Our previous studies did not establish female gender as a predictor of post-herpetic neuralgia [15]. Similarly, Dworkin and Schmader [45] did not find sex differences to be associated with the various aspect of herpes zoster, with the only exception being the intensity of acute pain which is higher in women than in men, as also confirmed by us previously [46]. It is conceivable that the earlier reported association between gender and long-term pain may have been a consequence of the fact that more women were in the higher age strata [47]. Also, among the 85 PHN subjects, who have been enrolled in the present study, there were more females than males as indeed is observed in the general population [15].

It is a matter of fact, however, that the median duration of pain before going to the pain clinic, which in the present study was equal to 9 months (range 6-150) in the total population sample, was found to be longer for women than men, i.e. 12 months (range: 6-150) and 6 months (range: 6-72), respectively, approaching the statistical significance and raising the question of gender differences with regard to the PHN.

Interestingly, a recent study shows that the incidence of CNS disease caused by alphaherpesvirus (VZV, HSV-1 and HSV-2) is associated with gender and its influence varies over the lifetime [48]. Starting from the observation that the susceptibility to and the severity of several viral infections is higher in men than in women [48], the Authors suggest that heightened antiviral responses typical of female gender, although effective for rapid virus clearance, can result in chronic/inflammatory pathologies, if excessively high or prolonged [49]. Their suggestion is based on the finding that compared to females, peripheral blood mononuclear cells from males produce lower amounts of IFN-α and higher amounts of the immunosuppressive cytokine IL-10 following stimulation with different toll-like receptors (TLRs) ligands or viruses, and some of these differences related with plasma levels of sex hormones [49]. Should this hypothesis be tested in a sufficiently large sample of patients with PHN, it could be evidence in favor of those who argue that protracted pain after HZ occurs either as a result of a severe neuronal injury at the time of zoster onset and/or a failure to recover normal neuronal function following an abnormal host’s inflammatory response [44],[50],[51].

Although current guidelines recommend antiviral treatment within 72 hours from the rash onset in individuals aged over 50 years, with the aim of reducing the incidence and duration of PHN, only around one half of our PHN patients had received appropriate treatment. However, when considering that, as reported recently, 78.4% of zoster patients receive antiviral therapy in Italy [52], the herein reported results confirm that antiviral therapy of acute zoster is absolutely effective although not completely satisfactory in the prevention of PHN. Consistently, the group of our PHN patients who had been treated with antivirals during acute zoster, showed a reduced risk of higher pain during the follow-up and a significantly lower percentage of individuals with residual neuropathic pain at T12, compared with the untreated groups of PHN patients. Although the correlation is based, at least in part, on anamnestic data, which represents a possible bias, its meaning is quite clear. These findings potentially suggest less nerve damage [53], but can also be explained by the variability of clinical response and even more importantly by the efficacy of pain treatments. Therefore, these results need further confirmation on larger, prospective and randomized studies.

Quality of life is dramatically reduced in patients with long-lasting PHN. Consistently, PCS and MCS scores of our patients at the time of enrollment in the study were found to be sharply lower than those of a large Italian population sample of healthy individuals, who were used as an historical age-matched control. As expected, not only the MCS scores but even more the PCS scores of our patients varied as a function of the patients’ age and were found to be lower in older individuals than in the younger ones. Also, it has to be emphasized that treatments used for pain relief seemed to have worked in all the patients enrolled in the study regardless of age groups or gender. This fact outlines the importance of a systematic and specialized approach to the problem of chronic pain in PHN patients. The nature of neuropathic pain in PHN is variable, so that it may be described as continuous or paroxysmal, evoked or spontaneous, burning or lancinating, and be associated with a range of other sensory abnormalities in the skin [54]. This variability in symptoms could imply that a variety of different pain mechanisms might be operating in different patients with PHN or in the same patient at different time points [54]. Therefore, treatment protocols should be optimised for individual patients on the basis of symptoms or even mechanisms [36]. Actually, no substantial evidence base exists to relate specific sets of symptoms or signs to the efficacy of specific drugs, and no simple validated methods exist to determine which neuropathic pain mechanism(s) may be operating in a single patient. Moreover, no single treatment has been shown to be completely effective for all sufferers of PHN, and combinations of analgesic drugs are usually required to achieve at least a partial relief of pain [36]. Tricyclic antidepressants, gabapentinoids and strong opioids are effective but are also associated with systemic adverse events that may limit their use in many patients, most notably those with significant medical comorbidities or advanced age [55]. Also, compliance of patients with treatment is an important factor in the clinical effectiveness of therapies, and a major factor governing compliance is withdrawal due to side effects (major harm).

Finally, our results do not allow us to confirm a clear relationship between the positivity/persistence of VZV viremia and PHN, since only 5 out of the 50 available blood samples from the PHN patients in the study were found positive for VZV DNA search. Other reports show comparatively higher percentages of VZV DNA-positivity in blood or saliva of patients affected by HZ and/or PHN [18],[56]. It is worth mentioning, however, that searching for VZV DNA in blood of our PHN patients was performed only once in the course of the study. A possible reason for the discrepancy of the PCR results may be also related to the sensitivity of the different methods for VZV-DNA detection in blood. For VZV, as well as for other herpes viruses, a variety of methods, techniques and protocols have been used to measure viral load at many institutions [57]. However, because each institution monitors VZV load with its own “homebrew” system, detection techniques and viral load estimation values have not been standardized and results vary between different laboratories [56],[57].

It seems that up to date, in spite of the large body of evidence available in the literature, there is still a need to gain new insights on the mechanism/s underlying PHN pathogenesis, which at present remains not fully elucidated. In detail, whether PHN pathogenesis is due to persistent productive virus infection in ganglia or to neuronal cell damage (i.e. altered excitability of ganglionic or even spinal cord neurons), is still controversial [58]-[67]. The possibility that protracted pain after zoster occurs either as a result of severe neuronal injury at the time of zoster onset or a failure to recover normal neuronal function [29],[30] is sustained by the very recent demonstration that there is only a modest recovery of sensory function and no anatomic recovery despite pain resolution in late follow-up of a group of PHN patients [44]. Further studies aimed at deepening our knowledge on PHN pathogenesis are currently in progress in our laboratories.

## Conclusions

We report that patients who still have significant neuropathic pain six months after acute zoster present a high probability of suffering from chronic pain in the subsequent months/years. Antiviral therapy used within 72 hours from the rash onset reduces the risk of higher VAS in the follow-up. Current strategies of analgesic therapy are effective to achieve relief of pain in PHN patients, but they are burdened with heavy and undesirable side effects. PHN represents a major public health problem. In spite of the large body of evidence available in the literature, there is still a need to gain new insights on chronic pain, its burden and the efficacy of available treatments.

## Authors’ original submitted files for images

Below are the links to the authors’ original submitted files for images. Authors’ original file for figure 1

## Abbreviations

PHN: Post-herpetic neuralgia
HZ: Herpes zoster
VZV: Varicella-zoster virus
PCR: Polymerase chain reaction
DN4 questionnaire: Douleur Neuropathique en 4 Questions
PCS: Physical Health Composite Scale
MCS: Mental Health Composite Scale
