# Supplementary figures and images for: One-year follow-up of patients with long-lasting post-herpetic neuralgia
Source: BMC Infect Dis. 2014 Nov 1;14:556. doi: 10.1186/s12879-014-0556-6 (PMC4226872; doi:10.1186/s12879-014-0556-6)

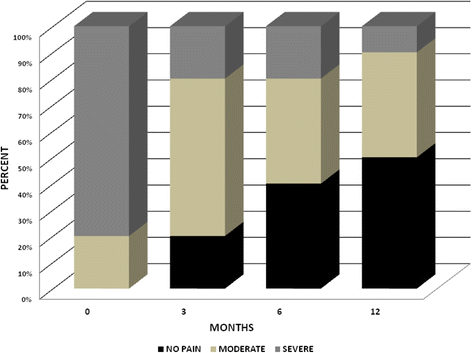

Supplement: Supplementary file 1 — Authors’ original file for figure 1 [file 12879_2014_556_MOESM1_ESM.gif]
